# Genome Sequences of 12 Phages That Infect Klebsiella pneumoniae

**DOI:** 10.1128/MRA.00024-20

**Published:** 2020-04-16

**Authors:** Trever L. Thurgood, Ruchira Sharma, Jackson J. Call, Joshua D. Chronis, Daniel D. Dawson, Zachary K. Finnegan, Kent W. Foster, Tyler Meek, Emily Potts, Michael R. Sirrine, Alex D. Atkinson, Jacob D. Fairholm, Yoga A. Handoko, Kai Li Ong, Olivia B. Tateoka, Daniel K. Arens, Liam Johnson, Jared L. Kruger, Emily Loertscher, Daniel W. Thompson, Jamison K. Walker, Richard A. Robison, Sherwood R. Casjens, Julianne H. Grose

**Affiliations:** a Department of Microbiology and Molecular Biology, Brigham Young University, Provo, Utah, USA; b Department of Agrotechnology, Satya Wacana Christian University, Salatiga, Central Java, Indonesia; c School of Biological Sciences, University of Utah, Salt Lake City, Utah, USA; d Division of Microbiology and Immunology, Department of Pathology, School of Medicine, University of Utah, Salt Lake City, Utah, USA; Queens College

## Abstract

Klebsiella pneumoniae is a pathogen responsible for significant proportions of nosocomial and health care-associated infections and is known to acquire multiple antibiotic resistance genes. Here, we announce the full genome sequences of 12 K. pneumoniae bacteriophages from samples collected in wastewater treatment facilities across the western United States.

## ANNOUNCEMENT

Klebsiella pneumoniae is a pathogenic member of the *Enterobacteriaceae* family that is responsible for a significant proportion (>10%) of hospital-acquired infections annually, as well as many community-acquired infections in the United States (3 to 5%) (1). K. pneumoniae is also known to be involved in the dissemination of a major class of carbapenemase genes (K. pneumoniae carbapenemases [KPCs]), which has contributed to the global spread of bacterial antibiotic resistance (2, 3). Classic treatments for K. pneumoniae infections are losing efficacy in the face of rising rates of antibiotic resistance. Therefore, the study of alternative treatments such as bacteriophage therapy could be beneficial in the future.

Here, we report the complete genome sequences of 12 K. pneumoniae bacteriophages isolated from wastewater in the western United States. All phages were propagated on Klebsiella pneumoniae ATCC 13883. Phages were amplified from enrichment cultures using LB medium at 37°C, plated on LB top agar at 37°C, and purified through a minimum of three successive single-plaque isolations (4). Phage genomic DNA was isolated from high-titer lysates using the phage DNA isolation kit from Norgen Biotek (Canada). The Illumina TruSeq DNA Nano kit was used for genomic library preparation with unique barcodes, followed by sequencing on the Illumina HiSeq 2500 platform (250-bp paired-end reads) at the Brigham Young University DNA Sequencing Center (Provo, UT). All contigs were assembled *de novo* using Geneious (5) version R11 and were annotated using DNA Master (6) and GeneMarkS (7) gene prediction software; all software was used with default settings. These 12 phages circularized upon assembly, and base pair 1 was called by alignment with the closest published phage relative that was reported as a complete genome, using BLASTn (8).

The 12 phage genomes can be placed into three previously established *Caudovirales* clusters, or groups of phages having homology over >50% of the genome (9, 10), according to our previous cluster definitions for *Enterobacteriaceae* phages (11). The largest cluster, populated by vB_KpnS_Domnhall, vB_KpnS_IMGroot, vB_KpnS_KingDDD, vB_KpnS_Call, vB_KpnS_SegesCirculi, vB_KpnS_Alina, and vB_KpnS_Penguinator, shows at least 85% average nucleotide identity (ANI) (as determined by Kalign [12]) among all seven *Siphoviridae* phages (average genome size, 52,075 ± 1,219 bp), which are T1-like (11) phages. The second largest cluster consists of three T7-like (11) podovirus phages, vB_KpnP_Sibilus, vB_KpnP_NahiliMali, and vB_KpnP_Emp27, the latter of which represents its own subcluster, sharing 63% ANI with the former two phages, which share 92% ANI with each other (average genome size, 39,442 ± 790 bp). The third cluster consists of one T4-like (11) *Myoviridae* phage, vB_KpnM_Potts1 (genome size, 169,384 bp). Phage vB_Kpn_Chronis is an unclassified temperate phage, with close relatives in many K. pneumoniae genomes, that forms a new cluster in the lambda-like supercluster (11). The division of these 12 phages into four clusters is consistent with the classifications outlined by the International Committee on Taxonomy of Viruses (ICTV) (13) (Table 1). T1-like and T4-like phages have been shown previously to package DNA by a headful mechanism and T7-like phages through direct terminal repeats, which is consistent with the apparently circular genomes achieved during phage assembly (14, 15).

**TABLE 1:** Basic properties and accession numbers of 12 K. pneumoniae phages

Phage name	GenBank accession no.	SRA accession no.	Total no. of reads	Fold coverage (range [avg read depth])	Genome length (bp)	No. of ORFs^*a*^	Taxonomy^*b*^	GC content (%)^*c*^
vB_KpnP_Emp27	MN013074	SAMN13072788	3,139	183–430 (281)	38,603	45	A	50.7
vB_KpnS_Domnhall	MN013075	SAMN12752290	441,095	41–250 (219.1)	54,438	90	W	51.6
vB_KpnS_IMGroot	MN013076	SAMN13155540	338,553	452–1,559 (988.7)	52,866	88	W	51.3
vB_KpnS_KingDDD	MN013078	SAMN13072790	35,976	110–357 (172)	51,562	83	W	51.6
vB_KpnS_Call	MN013079	SAMN13228337	208,987	630–1,495 (899.1)	51,487	82	W	51.5
vB_KpnS_SegesCirculi	MN013080	SAMN13228338	365,333	1,123–5,463 (1,764)	50,713	80	W	51.1
vB_KpnM_Potts1	MN013081	SAMN12752291	92,223	183–430 (281)	169,384	298	T	40.7
vB_KpnP_Sibilus	MN013082	SAMN13072791	45,438	2–287 (170.3)	40,171	53	A	51.2
vB_KpnS_Alina	MN013083	SAMN13072792	22,399	44–199 (99.5)	51,780	83	W	51.6
vB_KpnP_NahiliMali	MN013085	SAMN13072794	45,628	117–732 (173.7)	39,556	52	A	51.2
vB_Kpn_Chronis	MN013086	SAMN13072795	93,145	2–30 (14.6)	45,702	73	P	52.3
vB_KpnS_Penguinator	MN013087	SAMN12752292	11,098	5–43 (21.6)	51,678	87	W	51.5

a ORFs, open reading frames in the current annotation, including 8 tRNAs for vB_KpnM_Potts1 and 1 tRNA for vB_Kpn_Chronis.

b The following abbreviations are used for taxonomy, which is provided by whole-genome BLASTN (9) at >95% identity for species taxonomy and >50% identity for genus taxonomy, as recommended by the Bacterial and Archaeal Viruses Subcommittee of the ICTV: A, *Podoviridae*, *Autographivirinae*, unclassified *Teseptimavirus*; W, *Siphoviridae*, *Tunavirinae*, *Webervirus*; T, *Myoviridae*, *Tevenvirinae*, unclassified *Tevenvirinae*; P, unclassified *Podoviridae*. All 12 phages belong to the superkingdom of viruses and the order *Caudovirales*.

c GC content for the genome.

### Data availability.

The accession numbers for all 12 phages are found in Table 1.
